# Effects of liver-stage clearance by Primaquine on gametocyte carriage of *Plasmodium vivax* and *P*. *falciparum*

**DOI:** 10.1371/journal.pntd.0005753

**Published:** 2017-07-21

**Authors:** Rahel Wampfler, Natalie E. Hofmann, Stephan Karl, Inoni Betuela, Benson Kinboro, Lina Lorry, Mariabeth Silkey, Leanne J. Robinson, Ivo Mueller, Ingrid Felger

**Affiliations:** 1 Swiss Tropical and Public Health Institute, Basel, Switzerland; 2 University of Basel, Basel, Switzerland; 3 Divison of Population Health and Immunity, The Walter and Eliza Hall Institute of Medical Research, Parkville, Australia; 4 Department of Medical Biology, University of Melbourne, Victoria, Australia; 5 Vector Borne Diseases Unit, PNG Institute of Medical Research, Goroka, Papua New Guinea; 6 Centre for Biomedical Research, Burnet Institute, Melbourne, Australia; 7 Malaria Parasites & Hosts Unit, Institut Pasteur, Paris, France; Johns Hopkins Bloomberg School of Public Health, UNITED STATES

## Abstract

**Background:**

Primaquine (PQ) is the only currently licensed antimalarial that prevents *Plasmodium vivax* (*Pv*) relapses. It also clears mature *P*. *falciparum* (*Pf*) gametocytes, thereby reducing post-treatment transmission. Randomized PQ treatment in a treatment-to-reinfection cohort in Papua New Guinean children permitted the study of *Pv* and *Pf* gametocyte carriage after radical cure and to investigate the contribution of *Pv* relapses.

**Methods:**

Children received radical cure with Chloroquine, Artemether-Lumefantrine plus either PQ or placebo. Blood samples were subsequently collected in 2-to 4-weekly intervals over 8 months. Gametocytes were detected by quantitative reverse transcription-PCR targeting *pvs25* and *pfs25*.

**Results:**

PQ treatment reduced the incidence of *Pv* gametocytes by 73%, which was comparable to the effect of PQ on incidence of blood-stage infections. 92% of *Pv* and 79% of *Pf* gametocyte-positive infections were asymptomatic. *Pv* and to a lesser extent *Pf* gametocyte positivity and density were associated with high blood-stage parasite densities. Multivariate analysis revealed that the odds of gametocytes were significantly reduced in mixed-species infections compared to single-species infections for both species (OR_Pv_ = 0.39 [95% CI 0.25–0.62], OR_Pf_ = 0.33 [95% CI 0.18–0.60], p<0.001). No difference between the PQ and placebo treatment arms was observed in density of *Pv* gametocytes or in the proportion of *Pv* infections that carried gametocytes. First infections after blood-stage and placebo treatment, likely caused by a relapsing hypnozoite, were equally likely to carry gametocytes than first infections after PQ treatment, likely caused by an infective mosquito bite.

**Conclusion:**

Pv relapses and new infections are associated with similar levels of gametocytaemia. Relapses thus contribute considerably to the Pv reservoir highlighting the importance of effective anti-hypnozoite treatment for efficient control of Pv.

**Trial registration:**

ClinicalTrials.gov NCT02143934

## Author summary

*Plasmodium vivax* (*Pv)* mainly affects Asia, Central and South America as well as Ethiopia. In Papua New Guinea (PNG) *Pv* prevalence is among the highest worldwide. The biggest challenge for the control of *Pv* infections is the formation of dormant liver stages, which have the ability to relapse and cause disease even after successful clearance of asexual stages in the blood circulation. Primaquine is the only licensed drug that is able to prevent *Pv* relapses. A randomized treatment-to-reinfection cohort in Papua New Guinean children permitted permitted the study of *Pv* and *P*. *falciparum* gametocyte carriage after radical cure with Primaquine and to investigate the contribution of *Pv* relapses to transmission. We found that most gametocyte carriers in this study were detected in asymptomatic infections and that relapses and new infections are associated with similar *Pv* gametocyte production. These are strong arguments emphasizing the importance of sensitive detection and early treatment of asymptomatic and submicroscopic Plasmodium *spp*. infections and of anti-hypnozoite treatment for an effective control of *Pv*.

## Introduction

Primaquine (PQ) is the only currently licensed drug for preventing *Plasmodium vivax* (*Pv*) relapses [1], and also the only effective drug against mature gametocytes of *P*. *falciparum* (*Pf*) [2,3]. Since 2012, the World Health Organization recommends a single dose of PQ for treatment of *Pf* infections with the aim to reduce post-treatment *Pf* gametocyte carriage and thus the potential for onward malaria transmission [4].

Gametocyte development as well as morphology differs considerably between *Pv* and *Pf* [5]. *Pv* gametocytes mature rapidly and are detectable in the peripheral blood as early as two or three days following detection of blood-stage parasites by qPCR or light microscopy (LM), respectively [6,7]. In contrast, *Pf* gametocytes sequester for 7–10 days in the bone marrow before being released into the blood circulation [8], where they are observed by LM 10–15 days after the first detection of asexual parasites [9]. Gametocytes were observed in symptomatic *Pv* episodes at higher frequency compared to *Pf* episodes, despite 10-fold lower *Pv* blood-stage densities compared to *Pf* [10]. After drug treatment, *Pv* gametocytes are cleared within days after clearance of blood-stage infections in contrast to *Pf* gametocytes, which circulate over 3 weeks following successful blood-stage clearance [6,9,11,12]. Altogether the published data suggests that *Pv* infections produce proportionally higher gametocyte densities than *Pf* infections (at the same levels of asexual parasitaemia), and that *Pv* gametocytes mature more rapidly [9,12–14].

Not much is known about gametocyte production in primary *Pv* infections versus relapses from activated hypnozoites, mainly because in endemic settings it is impossible to distinguish both sources of infection. Our previous work in Papua New Guinea (PNG) showed that relapsing *Pv* infections contributed 73% of the gametocyte carriage [15]. A study in Thailand and Indonesia reported that densities by LM of *Pv* blood-stage parasites and gametocytes were similar in new infections and relapses [16]. Both studies indicated the need for efficient treatment of the hypnozoite reservoir for reducing *Pv* transmission [15,16].

A challenge in studying the investment of *Pv* infections in gametocytogenesis is the generally low and often submicroscopic density of asexual parasites and gametocytes. In addition, scarce *Pv* gametocytes can easily be misclassified by LM due to their resemblance to late trophozoites [17]. Investigating gametocyte production of *Pv* infections hence requires sensitive and specific molecular methods. For *Pf*, studying gametocytes by LM is more feasible because of the distinct crescent-shaped morphology of gametocytes and generally higher parasite densities; however also for *Pf*, molecular methods are crucial for studying gametocytes in low-density *Pf* infections. Molecular detection of gametocytes usually targets transcripts of the *Pf* or *Pv* 25 kDa ookinete surface antigen precursor (*pfs25* or *pvs25*, respectively) [18,19], which are highly expressed in mature gametocytes [20,21]. Expression of the *pfs25* transcripts is mainly female specific, hence male gametocytes are detected to a much lower extend by *pfs25-*based assays [22]. Female gametocytes are generally over-represented in peripheral blood samples with about 3.5 female per each male gametocyte [23,24]. It can therefore be estimated that *pfs25* RT-qPCR assays detect approx. 70% of the total number of gametocytes. Both *pfs25* and *pvs25* quantitative reverse transcription PCR (qRT-PCR) or nucleic acid sequence-based amplification (NASBA) can detect as few as 1 *Pf* gametocyte or 10 *Pv* gametocytes per 50 μl blood and are therefore up to 50x more sensitive than LM [18,19,25].

This study investigated gametocyte dynamics of *Pf* and *Pv* infections in school-aged PNG children after randomized treatment with blood-stage antimalarials plus PQ or placebo. This trial design permitted an evaluation of the contribution of hypnozoites to *Pv* infection parameters by comparison of two treatment arms. PQ treatment for clearance of hypnozoites reduced the risk of recurrent *Pv* blood-stage infection by 82% [95% CI 0.75–0.86], the risk of a *Pv* episode by 75% [95% CI 0.49–0.89], and the incidence of *Pv* gametocytes by 73% [95% CI 0.62, 0.81][15]. Another study conducted in Thailand and Indonesia revealed that density of gametocytes over time followed that of asexual parasites [16]. However, the Thai-Indonesian study depended on the presentation of patients at a health facility upon occurrence of a clinical episode and did not use molecular methods to detect submicroscopic asexual parasites or gametocytes [16]. Our initial report on the PNG cohort study is now extended to address the following questions: (i) what are risk factors for *Pf* and *Pv* gametocyte carriage? (ii) does gametocytaemia differ between *Pv* new infections and relapses? And (iii) does PQ treatment exert a long-term effect on *Pf* and *Pv* gametocytaemia?

## Methods

### Study design

The study was conducted in 2009 to 2010 in the Albinama area, East Sepik province, in PNG. A detailed study protocol has been published previously [15]. In brief, 504 children aged 5 to 10 years were randomized to two treatment arms and completed directly observed treatment (DOT) with a 3-day dose of Chloroquine (CQ), a 3-day dose of Artemether-Lumefantrine (AL) and either 20 doses of PQ (per day: 0.5 mg/kg) or placebo over four weeks. Children were screened for G6PD deficiency by using a visual colorimetric method (G6PD Assay Kit WST-Dojindo Co., Japan). Venous blood samples were collected at enrolment (prior to treatment) and 3 days after the final dose of DOT. The latter date represented day 0 of follow-up. Finger-prick samples were taken every two weeks for the first 3 months and monthly for the remaining 5 months of follow-up. Symptomatic children detected during follow-up were treated with a 3-day course of AL after confirming *Plasmodium* infection by rapid diagnostic test (RDT, CareStart Malaria pLDH/HRP2 Combo, AccessBio, USA).

#### Ethics statement

The study received ethical clearance by the PNG Institute of Medical Research (IMR) Institutional Review Board (0908), the PNG Medical Advisory Committee (09.11), the Ethikkommission beider Basel (237/11) and was registered on ClinicalTrials.gov NCT02143934. A parent or guardian of every child participant provided written informed consent for their participation.

### Detection of blood-stage parasites and gametocytes

All blood samples collected were examined by LM and quantitative PCR (qPCR). Blood slides were examined by at least two independent microscopists and declared parasite negative only after examination of 200 thick-film fields [15]. Parasite DNA was extracted from 100–150 μl blood cell pellet using the FavorPrep 96-well genomic DNA extraction kit (Favorgen, Taiwan) and analyzed for *Pf* and *Pv* positivity by *18S rRNA* qPCR [15,19]. All *Pv* and *Pf* qPCR positive samples were genotyped using markers *Pv-msp1*F3 and *Pf-msp2*, respectively, following previously published protocols [26,27].

RNA was extracted from all samples positive in *Pf* or *Pv* qPCR. RNA was extracted using the RNEasy 96 kit (Qiagen, Switzerland) as described previously [19] from 50μl whole blood spotted on filter papers that had been air-dried and stored in TRIzol reagent (Life Technologies, Switzerland). Gametocyte-specific transcripts were detected by *pfs25* or *pvs25* qRT-PCR [19] in all RNA samples for which the corresponding DNA sample had been positive by species-specific qPCR.

### Statistical analysis

Children were censored on the last visit before two consecutively missed scheduled follow-up visits [15]. Comparison of LM-positive versus submicroscopic infections, and symptomatic versus asymptomatic infections was performed with 5019 samples from the follow-up period for which LM data was available. A clinical malaria episode was defined as fever (axillary temperature >37.5°C and/or fever reported in previous 2 days) and the presence of *Plasmodium spp*. parasites by LM. Differences in proportions were tested for statistical significance using the McNemar X^2^ test with continuity correction. To achieve normal distribution, qPCR densities were expressed as log_10_-transformed *18S rRNA* genomic copies/μl blood for asexual parasites, and log_10_-transformed *pfs25* or *pvs25* transcripts/μl blood for gametocytes. Correlation between microscopic parasite counts and molecular methods was tested by Kendall’s rank sum test on log_10_ transformed data. Geometric means of densities were calculated. Differences in densities of asexual or sexual-stage parasites were tested for statistical significance using Welch’s Two-sample t-test.

Negative binomial regression models were used to calculate the incidence rate of *Pv* and *Pf* gametocyte positivity as previously described [15]. Gametocyte positivity during follow-up was modeled using binomial generalized estimating equations (GEE) with logit link using an exchangeable correlation matrix to account for repeated measures by child. Log_10_-transformed blood-stage parasite density and gametocyte density during follow-up were modeled using Gaussian GEEs with log link using an exchangeable correlation matrix. Linear fit for log_10_-transformed blood-stage parasite density was previously analyzed and considered adequate for both species (S1 Text). All Models were back-selected. Statistical analyses were conducted using R version 3.1.1 [28] or STATA version 14.

## Results

### Gametocyte positivity and density in submicroscopic infections

**Fig 1:**
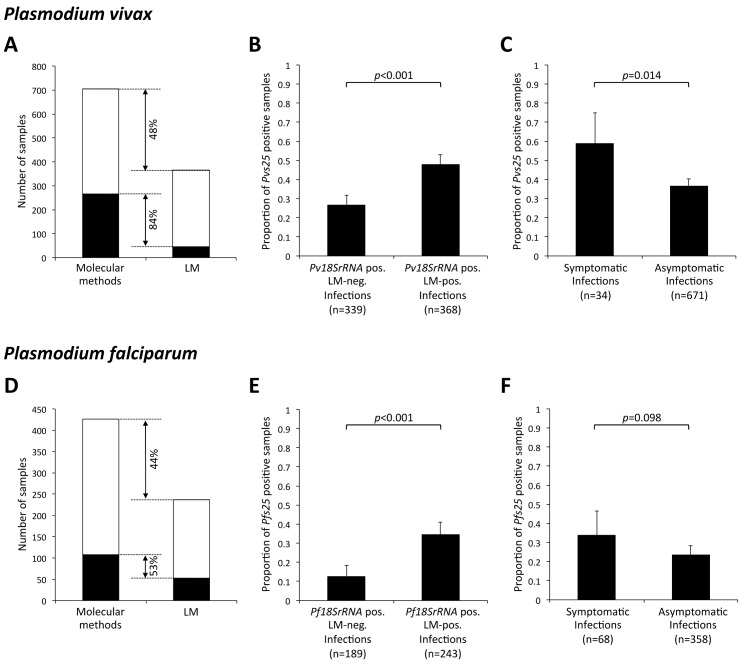
*Pv* (top) and *Pf* (bottom) gametocyte positivity among 5019 follow-up samples. (**A, D**) Detection of blood stage parasites and gametocytes by LM and molecular methods, using *pv18S* or *pf18S rRNA* qPCR for detection of blood-stage parasites and *pvs25* or *pfs25* qRT-PCR for detection of gametocytes. Black: gametocyte positive samples. White: parasite positive samples without gametocytes. (**B, E**) Proportion of gametocytes positives (by molecular methods) in submicroscopic and LM-positive samples. (**C, F**) Proportion of gametocyte positives (by molecular methods) in symptomatic and asymptomatic infections. Error bars indicate 95% confidence intervals by X^2^ distribution.

Molecular methods were superior to LM especially for detection of gametocytes but also for blood-stage parasites (Fig 1). By LM *Pv* gametocytes were detected in only 44 out of 366 *Pv* positive samples (12%), whereas by molecular detection 265 out of 705 *Pv* samples (38%, p<0.001) were gametocyte-positive. *Pf* gametocyte rates by LM were 21% (52/237) and by qRT-PCR 25% (107/426). 84% [CI_95_: 79–88%] and 53% [CI_95_: 43–63%] gametocytaemia was submicroscopic, for *Pv* and *Pf* respectively (Fig 1A and 1D). In one *Pv* and two *Pf* samples gametocytes were detected by LM but not by qRT-PCR, indicating most probably RNA degradation. For *Pv* infections, a late-stage trophozoite can be misread as a gametocyte, however LM slides of this study were read by three independent microscopists. Overall, gametocyte densities by LM and by molecular methods were significantly correlated in samples positive by both methods (Kendall’s tau test, pvs25: tau = 0.24, *p*-value = 0.037, *pfs25*: tau = 0.23, *p*-value = 0.027). Due to the low sensitivity of LM in gametocyte detection, all further results presented here derive from molecular gametocyte detection.

Significantly more *Pv* infections carried qRT-PCR detectable gametocytes compared to *Pf* (38% vs. 25%, *p*<0.001). Microscopically patent infections of both species carried gametocytes more often than submicroscopic infections (*Pv*: 48% vs. 27%, *Pf*: 35% vs. 13%, *p*<0.001, Fig 1B and 1E). Similarly, gametocyte-specific transcript numbers were significantly higher in LM-positive than LM-negative samples for both species (S1 Fig).

### Gametocyte positivity and density in symptomatic versus asymptomatic infections

During the follow-up period, 34 *Pv* episodes and 68 *Pf* clinical episodes were observed. The proportion of gametocyte carriers was 22% higher in clinical episodes compared to asymptomatic *P*. *vivax* infections (59% vs. 37%, *p* = 0.014, Fig 1C). For *P*. *falciparum*, a similar trend was observed but did not reach statistical significance (34% vs. 23%, *p* = 0.098, Fig 1F). However, due to a much higher number of asymptomatic infections than clinical episodes, the overwhelming majority of *Pv* and *Pf* gametocyte carriage (92% [CI_95_: 88–95%] and 79% [CI_95_: 69–86%]) occurred in asymptomatic children. *Pv* gametocyte densities showed the same trend as asexual densities in both clinical episodes and asymptomatic infections, but this was not the case for *Pf* (S2 Fig).

### The effect of PQ treatment on gametocytaemia during follow-up

**Fig 2:**
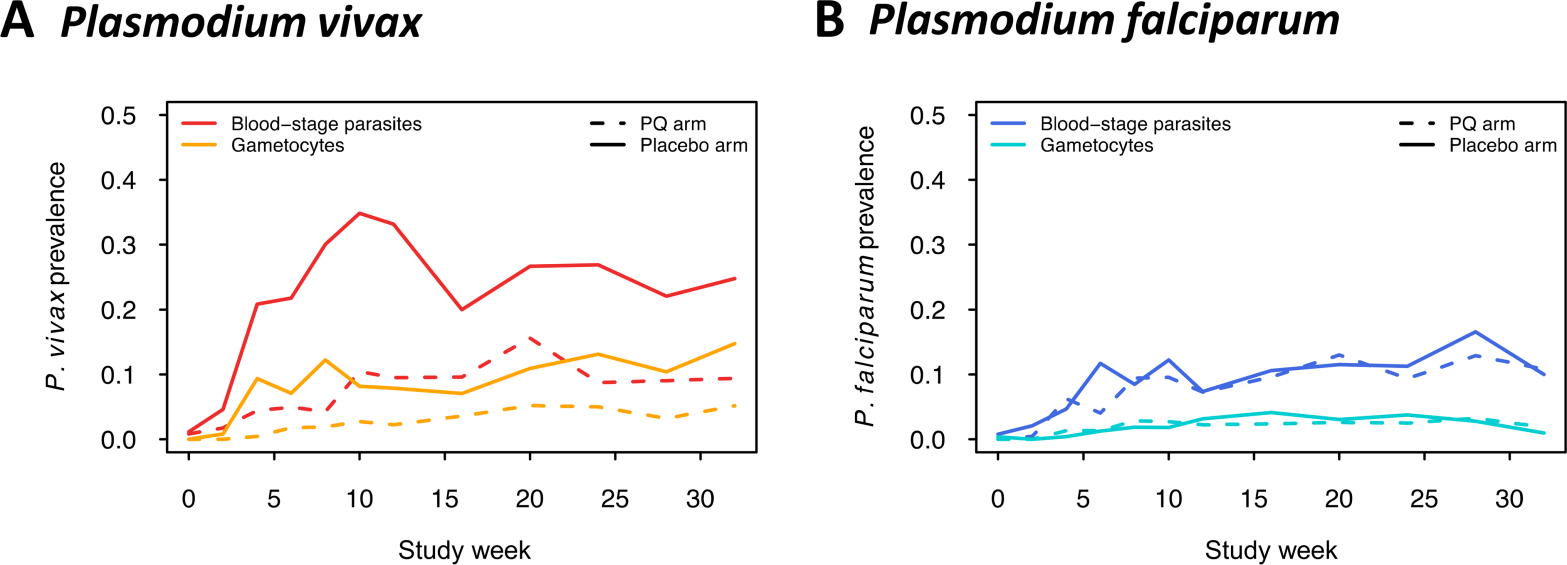
Prevalence of blood-stage parasites and gametocytes of *Pv* (A) and *Pf* (B) during follow-up by treatment arm.

*Pv* gametocyte prevalence increased steadily throughout the follow-up period and was on average almost 3-fold higher in the placebo arm than in the PQ arm, similar to patterns observed in *Pv* blood-stage parasite prevalence (*Pv* gametocytes median fold difference PL>PQ: 2.9 [IQR: 2.0–3.8], *Pv* blood-stages median fold difference PL>PQ: 2.8 [IQR: 2.2–4.2], Fig 2A). No difference in *Pf* gametocyte prevalence was observed between study arms (*Pf* gametocytes median fold difference PL>PQ 1.0 [IQR: 0.7–1.4], *Pf* blood-stages median fold difference PL>PQ: 1.1 [IQR: 0.9–1.3], Fig 2B).

**Fig 3:**
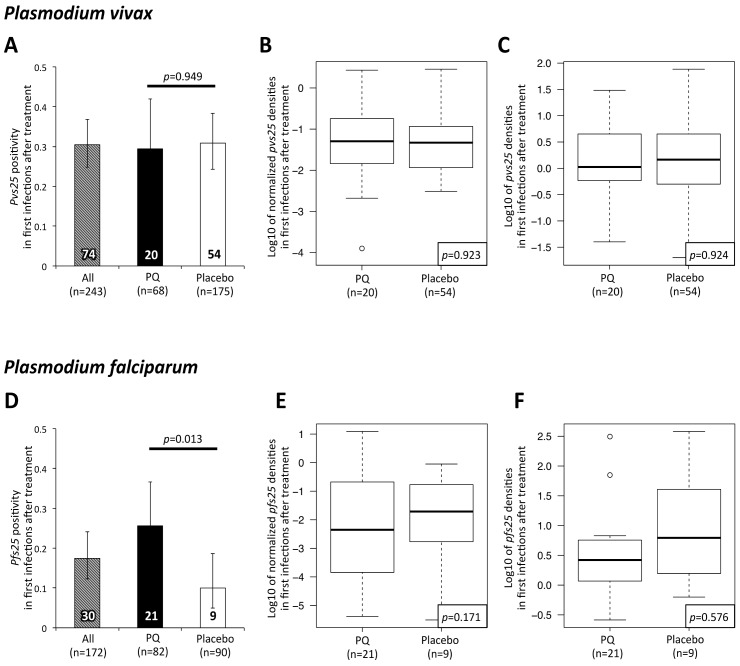
Gametocyte positivity and density in first *Pv* (top) and *Pf* (bottom) infections after treatment with blood-stage antimalarials alone (placebo) or blood-stage antimalarials plus PQ (PQ). (**A, D**). Proportion of *Pv* and *Pf* gametocyte carriers among first infections by treatment arm. Figures within the bars indicate absolute numbers of gametocyte-positive first infections following treatment. Error bars indicate 95% confidence intervals by X^2^ distribution. (**B, E**). Normalized *Pv* and *Pf* gametocyte densities in first infections by treatment arm. Densities were normalized by dividing *pvs25* or *pfs25* transcript numbers/μl by *Pv-* or *Pf-18S rRNA* copy numbers/μl. (**C, F**) Absolute *Pv* and *Pf* gametocyte densities in first infections by treatment arm. Densities are expressed as log_10_ of *pvs25* and *pfs25* transcripts/μl.

To assess in detail *Pv* gametocyte production in primary infections versus relapses, we compared gametocyte positivity and density in first infections after blood-stage plus placebo (first *Pv* infection either from relapse (80%80%) or infective bite (20%)) or blood-stage plus PQ treatment (first *Pv* infection always from infective bite) [15]. We also assessed subsequent *Pv* infections, i.e. all but the first parasite-positive sample per child, which in both arms can result from an ongoing infection, a relapsing hypnozoite or a new infection from a mosquito. First *Pv* re-infections after baseline treatment were equally likely to carry gametocytes in both treatment arms (PQ: 29% vs. Placebo: 31%, Fig 3A), and the same was observed for subsequent infections (PQ: 42% [CI_95_: 32–52%] vs. Placebo: 41% [CI_95_: 36–47%], *p* = 1). To investigate whether gametocyte densities were simply following the asexual densities or if other factors play a role, we compared absolute as well as normalized gametocyte densities. Gametocyte densities were normalized by dividing *pvs25* or *pfs25* transcript numbers/μl by *Pv-* or *Pf-18S rRNA* copy numbers/μl. Absolute and normalized *Pv* gametocyte densities did not differ between treatment arms in first infections (Fig 3B and 3C) nor in subsequent infections (S3 Fig).

We also investigated *Pf* gametocyte carriage by comparing *Pf* gametocyte positivity and density in first *Pf* infections after treatment. Significantly more *Pf* gametocyte carriers were observed among first infections in the PQ-arm compared to the placebo arm (PQ: 26% vs. Placebo: 10%, Fig 3D). No significant difference between trial arms was observed in subsequent samples (PQ: 30% [CI_95_ 22–39%] vs. Placebo: 31% [CI_95_ 23–40%], p = 0.961). *Pf* absolute and normalized gametocyte densities did not differ significantly between the treatment arms (Fig 3E and 3F, S3 Fig).

### Risk factors for gametocytes positivity and density

*Pv* gametocytes were detected more frequently (Table 1, OR for 1-log increase of density = 1.95, *p*<0.001) and in higher densities (Table 2, *p*<0.001) with increasing blood-stage parasite density. Apart from reducing the number of *Pv* positive samples during follow-up (S1 Table), PQ treatment had no further effect on *Pv* gametocyte positivity (Table 1).

**Table 1:** Multivariable predictors of *Pv* and *Pf* gametocyte positivity during follow-up.

	*Pv* gametocyte positive				*Pf* gametocyte positive			
	OR	95% CI		*p*-value	OR	95% CI		*p*-value
Blood-stage density (by qPCR), per 10x increase	1.95	1.47	2.58	<0.001	1.23	0.99	1.51	0.059
PQ treatment	1.03	0.68	1.58	0.878	1.21	0.78	1.90	0.395
Mixed *Pf*/*Pv* (by qPCR)	0.39	0.25	0.62	<0.001	0.33	0.18	0.60	<0.001
First infection	0.64	0.42	0.98	0.040	0.45	0.25	0.81	0.007
Days after DOT (ref:0–60)								
61–120	0.59	0.39	0.89		1.31	0.62	2.76	
121–180	1.48	0.91	2.42	<0.001	1.32	0.63	2.75	0.266
>180	2.54	1.47	4.39		0.73	0.32	1.67	
Constant	0.36	0.22	0.59	<0.001	0.29	0.11	0.77	<0.013

OR, odds ratio

DOT, directly observed treatment.

ORs were obtained using binomial generalized estimating equations with logit-link allowing for repeated visits by back-selection from the full model. The full model included fever, infection status at enrolment by qPCR (*Pf* or *Pv* positive), LLIN use (less than 100%), sex, village of residence, hemoglobin at baseline (>9 g/dl), age. No significant interaction of PQ treatment with days post DOT was detected.

**Table 2:** Multivariable predictors of *Pv* and *Pf* gametocyte density during follow-up.

	*Pv* gametocyte density			
	exp(β)	95% CI		*p*-value
Blood-stage density (by qPCR) per 10x increase	1.37	1.20	1.56	<0.001
PQ treatment	0.97	0.80	1.18	0.765
Mixed *Pf*/*Pv* (by qPCR)	0.73	0.59	0.90	0.003
Age	0.94	0.89	0.99	0.017
Days after DOT (ref: 0–60)				
61–120	1.08	0.86	1.35	
121–180	1.15	0.90	1.47	0.163
>180	1.30	1.03	1.64	
Constant	1.11	0.69	1.81	0.661

β, regression coefficient.

Coefficients were obtained using Gaussian generalized estimating equations with log-link by allowing for repeated visits and by back-selection from the full model. The full model included fever, infection status at enrolment by qPCR (*Pf* or *Pv* positive), LLIN use (less than 100%), sex, village of residence, hemoglobin at baseline (>9 g/dl), first infection. No predictors were associated with *Pf* gametocyte densities (S1 Text). No significant interaction of PQ treatment with days post DOT was detected.

In *Pv* positive samples, the odds of *Pv* gametocytes were 60% reduced and gametocyte densities were 30% lower in mixed *Pf*/*Pv* infections compared to single-species *Pv* infections (Table 1, *p*<0.001; Table 2 *p* = 0.003). The odds for *Pv* gametocyte carriage increased significantly over the whole follow-up period (Table 1 and Fig 2, *p*<0.001), and a 36% reduction on the odds of being gametocyte positive was observed in first *Pv* infections compared to subsequent infections (Table 1, *p* = 0.040). No other factors were associated with the odds for *Pv* gametocyte carriage during follow-up (Table 1). *Pv* gametocyte density, but not positivity, decreased with age (Table 2, *p* = 0.017) following the age trend in asexual parasites (Table 3, [29]).

**Table 3:** Multivariate predictors *Pv* and *Pf* blood-stage parasite density during follow-up.

	*Pv* blood-stage density				*Pf* blood-stage density			
	exp(β)	95% CI		*p*-value	exp(β)	95% CI		*p*-value
PQ treatment	1.04	0.93	1.15	0.505	0.85	0.68	1.06	0.143
Mixed *Pf*/*Pv* (by qPCR)	-	-	-	-	0.79	0.63	1.00	0.048
Fever	-	-	-	-	2.41	1.82	3.19	<0.001
Age	0.96	0.92	0.99	0.014	-	-	-	-
Days after DOT (ref: 0–60)								
61–120	0.94	0.83	1.07		0.93	0.69	1.25	
121–180	0.72	0.62	0.83	<0.001	0.89	0.62	1.25	<0.001
>180	0.51	0.44	0.59		0.59	0.43	0.80	
Constant	5.13	3.83	6.87	<0.001	16.37	12.37	21.66	<0.001

β, regression coefficient.

Coefficients were obtained using Gaussian generalized estimating equations with log-link by allowing for repeated visits and by back-selection from the full model. The full model included fever, infection status at enrolment by qPCR (*f*. or *v*. positive), LLIN use (less than 100%), sex, village of residence, hemoglobin at baseline (>9 g/dl), first infection. Non-associated predictors were shown by “-”in the respective line. No significant interaction of PQ treatment with days post DOT was detected.

As for *Pv* gametocytes, the odds for *Pf* gametocytes were 70% reduced in mixed *Pf*/*Pv* infections compared to single-species *Pf* infections (Table 1, *p*<0.001). As an effect of delayed *Pf* gametocyte maturation, gametocyte positivity was 55% lower in first *Pf* infections compared to subsequent infections (Table 1, *p* = 0.007). Other risk factors for *Pf* gametocytes were investigated, but none of the parameters tested was significant. The *Pf* gametocyte positivity was slightly higher in samples with high asexual densities, yet this association did not reach the 5% significance level (Table 1, OR for 1-log increase of density = 1.23, *p* = 0.059). In contrast to *Pv*, *Pf* gametocyte densities were not associated with any of the factors assessed (S1 Text). Fever was strongly associated with increasing blood-stage *Pf* parasitaemia (Table 3, OR = 2.41, *p*<0.001), but had no effect on gametocyte density.

Analysis of only subsequent infections showed similar results to the analysis of the entire follow-up period (S2 Table). Considering subsequent infections only, *Pv* gametocytes were reduced by 47% [13–68%] and *Pf* gametocytes were reduced by 63% [26–82%] in mixed-species infections compared to single-species infections (*Pv p-*value: 0.013, *Pf p-*value: 0.006, S2 Table). It was not possible to analyse the effect of mixed-species co-infection on gametocyte carriage in first positive samples following treatment due to very low sample size for either species.

## Discussion

This study represents a first detailed investigation of the contribution of *Pv* relapses to the infectious reservoir. The transmission potential attributable to relapses was estimated by comparing gametocyte positivity and density in children that had received either PQ or placebo treatment. A major finding was that *Pv* gametocytes were detected in equal proportions and equal density in *Pv* positive samples of both trial arms. In the PQ arm, the majority of *Pv* infections derived from new mosquito bites, while in the placebo arm 80% of infections were caused by relapsing hypnozoites [15]. Gametocyte densities as well as the proportion of gametocyte carriers concurred in both arms, thus indicating that new and relapsing infections produce gametocytes at equal rates. Similar conclusions were drawn from a study in south-east Asia, where *Pv* gametocyte densities and positivity had closely mirrored parasitaemia in both, clinical primary and recurrent infections [16]. Gametocyte production in relapses thus seems indistinguishable from that in new infections. This finding highlights the importance of anti-hypnozoite drugs to prevent relapses for an effective interruption of *Pv* transmission.

Sample storage in this cohort was not optimal for RNA preservation. Blood was spotted onto Whatman 3MM filter paper in the field, and stored at room temperature for up to 5 weeks until transferred into TRIzol reagent. This procedure was suboptimal compared to sampling in RNA-stabilizing reagents [19]. A more recent cross-sectional study in PNG employed sampling in RNAprotect Cell Reagent (Qiagen, Switzerland) and found gametocytes in 78% and 60% of *Pf* and *Pv* qPCR-positive samples in children aged 6–9 years [25]. Almost universal *Pv* gametocyte prevalence (95%) was found in Brazilian samples stored in liquid nitrogen [30]. The relatively low gametocyte positivity in this cohort was indicative of poor RNA quality, which likely resulted in a substantial underestimation of gametocyte rates. The gametocyte rate in the present study thus reflects a minimum prevalence. Because RNA quality and sample volume did not vary within the study, the comparative analyses of treatment arms and risk factors remain unaffected, even if these results need to be regarded as referring to infections with moderately high gametocyte densities.

The vast majority (>80%) of gametocyte carriers were asymptomatic for both species, and over 20% of *Pv* and over 30% of *Pf* gametocyte positive samples were submicroscopic. Although gametocyte densities were lower in submicroscopic infections compared to LM-positive infections for both species, they may nonetheless be potentially infective to mosquitoes. Mosquito feeding experiments have demonstrated that submicroscopic infections can infect mosquitoes, albeit at lower rates than microscopically patent infections, and thus contribute to onward transmission [31–35]. Our results highlight the importance of treating all malaria infections in the community, as asymptomatic individuals will not report themselves to health facilities and thus generally remain untreated and infectious for longer periods.

Co-infections with both species are common in PNG [36,37] including in this cohort, and interactions between co-infecting species in mixed infections have been investigated previously. However, these former studies focused on the asexual stages of *Pv* and *Pf* [38,39] or risk for clinical diseases [29,38,40] and did not address transmission stages. Gametocytes in the host are influenced by a complex interplay of parasite factors (such as stress response) and host factors (such as immunity), and this complexity is enhanced by a second co-infecting *Plasmodium* species. Our finding of significantly reduced gametocytes in mixed-species infections compared to single-species infections is a first indication of species interaction affecting the transmission stages. Confirmation of our results is required in other studies investigating *Plasmodium* species interactions with specific focus on the transmission stages.

In the first post-treatment *Pf* infections gametocytes were more frequently detected in the PQ arm than in the placebo arm. This is likely explained by the slower acquisition of new *Pv* infections in PQ-treated individuals, compared to a fast relapse rate in individuals retaining hypnozoites in the liver. Indeed, in the placebo arm 52% of first *Pf* infections carried a *Pv* co-infection as opposed to only 21% in the PQ arm. Accordingly, the multivariate analysis showed reduced odds of *Pf* as well as *Pv* gametocytes in mixed-species infections compared to single-species infections. In addition, a *Pf* co-infection reduced *Pv* gametocyte densities by half. A study in 0.5 to 5 year old PNG children with uncomplicated malaria confirmed that *Pv* gametocytaemia in *Pf/Pv* mixed infections was reduced compared to *Pv* single infections [41]. Moreover, a community study in PNG showed a lower proportion of *Pf* gametocyte carriers in *Pf*/*Pv* mixed infections compared to *Pf* single-species infections [25]. Similar findings had been reported from Thailand [42]. More longitudinal studies designed specifically to address gametocyte dynamics in mono- and mixed-species infections are needed to confirm potential cross-species interaction and its effect on sexual stage development.

### Conclusion

Onset and rate of *Pv* gametocyte production did not differ between relapses and primary infections. This is a strong argument for treatment policies and elimination strategies that support PQ treatment of all *Pv* infections. The vast majority of gametocyte carriers in this study were detected in asymptomatic infections, which suggests that sensitive detection and early treatment of asymptomatic and submicroscopic Plasmodium *spp*. infections may be crucial for an effective control of transmission. PQ treatment prevented relapses and thus reduced *Pv* gametocyte carriage by 73%. These and other *Plasmodium* species interactions that can substantially affect gametocyte production warrant further investigation.

## Supporting information

S1 Text Evaluation of the multivariate analysis (GEE models) of *P*. *vivax* and *P*. *falciparum*. (DOCX)

S1 Fig Gametocyte and overall parasite density in submicroscopic and LM-positive *Pv* (top) and *Pf* (bottom) infections. A. and C. *Pv* and *Pf* gametocyte densities were expressed as log10 of *pvs25* and *pfs25* transcripts/μl. B. and D. *Pv* and *Pf* parasite densities were expressed as log10 of *pv18S rRNA* and *pf18S rRNA* gene copies/μl. C. and E. *Pv* and *Pf* normalized gametocyte densities. Densities were normalized by division of *pvs25* and *pfs25* transcripts/μl by *pv18S rRNA* or *Pf18S rRNA* genomic copies/μl, respectively.
(DOCX)

S2 Fig Gametocyte and parasite density in symptomatic and asymptomatic *Pv* (top) and *Pf* (bottom) infections. A. and C. *Pv* and *Pf* gametocyte densities were expressed as log10 of *pvs25* and *pfs25* transcripts/μl. B. and D. *Pv* and *Pf* parasite densities were expressed as log10 of *pv18S rRNA* and *pf18S rRNA* gene copies/μl. C. and E. *Pv* and *Pf* normalized gametocyte densities. Densities were normalized by division of *pvs25* and *pfs25* transcripts/μl by *pv18S rRNA* or *Pf18S rRNA* genomic copies/μl, respectively.
(DOCX)

S3 Fig Gametocyte positivity and density in subsequent (i.e. not first) *P*. *vivax* (top) and *P*. *falciparum* (bottom) infections after treatment with blood-stage antimalarials alone (Placebo) or blood-stage antimalarials plus Primaquine (PQ). A. and C. Proportion of *P*. *vivax* and *P*. *falciparum* gametocyte carriers among subsequent infections by treatment arm. Figures within the bars indicate absolute numbers of gametocyte-positive subsequent infections following treatment. Error bars indicate 95% confidence intervals by X^2^ distribution. B. and E. Normalized *P*. *vivax* and *P*. *falciparum* gametocyte densities in subsequent infections by treatment arm. Normalization was done by dividing *pvs25* or *pfs25* transcript numbers/μl by *Pv-* or *Pf-18S rRNA* copy numbers/μl. C. and F. Absolute *P*. *vivax* and *P*. *falciparum* gametocyte densities in subsequent infections by treatment arm. Densities are expressed as log_10_ of *pvs25* and *pfs25* transcripts/μl.
(DOCX)

S1 Table Multivariate risk factors of *P*. *vivax* and *P*. *falciparum* asexual parasite positivity during follow-up. (DOCX)

S2 Table Multivariate risk factors of *P*. *vivax* and *P*. *falciparum* gametocyte carriage in subsequent infections during follow-up. (DOCX)
