# Supplementary material for: Effects of liver-stage clearance by Primaquine on gametocyte carriage of Plasmodium vivax and P. falciparum
Source: PLoS Negl Trop Dis. 2017 Jul 21;11(7):e0005753. doi: 10.1371/journal.pntd.0005753 (PMC5540608; doi:10.1371/journal.pntd.0005753)
Supplement: S1 Text — (DOCX) [file pntd.0005753.s001.docx]

# Effects of liver-stage clearance by Primaquine on gametocyte carriage of *Plasmodium vivax* and *P. falciparum*

***Wampfler et al. 2017***

**S1 TEXT**

**Evaluation of the multivariate analysis (GEE models) of *P. vivax*  and *P. falciparum.***

**A****B**
**Figure S1.1 Density distribution of *P. vivax*. (A) *Pv* blood-stage densities by genomic 18S rRNA copies/µl (log_10_). (B) *Pv* gametocytes densities by *pvs25* transcript copies/µl (log_10_). Log_10_ transformations of copies/µl show normal distribution.**

**A****B**
**Figure S1.2 Density distribution of *P. facliparum*. (A) *Pf* blood-stage densities by genomic 18S rRNA copies/µl (log_10_). (B) *Pf* gametocytes densities by *pfs25* transcript copies/µl (log_10_). Log_10_ transformations of copies/µl show almost normal distribution.**

**A****B**
**Figure S1.3 Scatterplot of *Pv* (A) and *Pf* (B) blood-stage vs. gametocyte density (log_10_).**

**Table S1.1 GEE Models for *P. vivax* gametocyte positivity with categorical (red) *Pv* blood-stage densities (genomic 18S rRNA copies/µl). NOTE:** Association of categorical *Pv* densities with *Pv* gametocyte positivity is almost linear.

| ***Pv* gametocyte positivity – categorical *Pv* density** | OR | 95% CI | | p-value |
| --- | --- | --- | --- | --- |
| Pv density (ref: log10 density <0) | | |  |  |
| 0-0.5 | 2.12 | 0.90 | 4.98 | <0.001 |
| 0.5-1 | 2.73 | 1.25 | 5.93 |  |
| 1-1.5 | 3.01 | 1.43 | 6.34 |  |
| 1.5-2 | 4.09 | 1.83 | 9.15 |  |
| >2 | 10.48 | 4.22 | 26.06 |  |
| PQ treatment | 1.03 | 0.67 | 1.57 | 0.905 |
| Mixed Pf/Pv | 0.40 | 0.25 | 0.63 | <0.001 |
| First Pv infection | 0.63 | 0.41 | 0.96 | 0.031 |
| Days post treatment (ref: 0-60) | | |  |  |
| 61-120 | 0.58 | 0.39 | 0.87 | <0.001 |
| 121-180 | 1.46 | 0.89 | 2.38 |  |
| >180 | 2.42 | 1.38 | 4.23 |  |
| Constant | 0.25 | 0.11 | 0.57 | 0.001 |

**Table S1.2 Category counts of the GEE Models for *P. vivax* gametocyte positivity with categorical *Pv* blood-stage densities (genomic 18S rRNA copies/µl). NOTE:** The increase of *Pv* gametocyte positivity with increasing *Pv* asexual density is almost linear.

| **Category counts** | *Pv* gametocyt*e* positivity | |  |
| --- | --- | --- | --- |
| *Pv* density category  log_10_(18S rRNA copyno) | 0 | 1 | Total |
| <0 | 74 | 36 | 110 |
| 0-0.5 | 59 | 38 | 97 |
| 0.5-1 | 140 | 95 | 235 |
| 1-1.5 | 183 | 134 | 317 |
| 1.5-2 | 58 | 61 | 119 |
| >2 | 28 | 52 | 80 |
| Total | 542 | 416 | 958 |

**Table S1.3 GEE Models for *P. vivax* gametocyte density (pvs25 transcripts/µl) with categorical (red) *Pv* blood-stage densities (genomic 18S rRNA copies/µl). NOTE:** Association of categorical *Pv* densities with *Pv* gametocyte densities is almost linear.

| ***Pv* gametocyte density – categorical *Pv* density** | OR | 95% CI | | p-value |
| --- | --- | --- | --- | --- |
| *Pv* density (ref: log10 density <0) | | | |  |
| 0-0.5 | 1.56 | 1.01 | 2.41 | <0.001 |
| 0.5-1 | 1.49 | 0.92 | 2.40 |  |
| 1-1.5 | 1.43 | 0.93 | 2.19 |  |
| 1.5-2 | 2.35 | 1.43 | 3.86 |  |
| >2 | 2.50 | 1.54 | 4.04 |  |
| PQ treatment | 0.95 | 0.79 | 1.16 | 0.648 |
| Mixed Pf/Pv | 0.72 | 0.58 | 0.90 | 0.003 |
| Age | 0.94 | 0.90 | 0.99 | 0.031 |
| Days post treatment (ref: 0-60) | | |  |  |
| 61-120 | 1.05 | 0.84 | 1.31 | 0.272 |
| 121-180 | 1.12 | 0.89 | 1.40 |  |
| >180 | 1.24 | 0.99 | 1.54 |  |
| Constant | 0.97 | 0.54 | 1.73 | 0.909 |

**Table S1.4 GEE Models for *P. falciparum* gametocyte positivity with categorical (red) *Pf* blood-stage densities (genomic 18S rRNA copies/µl). NOTE:** Association of categorical *Pf* densities with *Pf* gametocyte positivity is not very linear, but *Pf* density is anyways not significant neither as linear nor categorical. For simplicity, linear was chosen.

| ***Pf* gametocyte positivity – categorical *Pf* density** | OR | 95% CI | | | p-value |
| --- | --- | --- | --- | --- | --- |
| Pf density (ref: log10 density <1) | | |  | |  |
| 1-2 | 0.35 | 0.13 | | 0.94 | 0.089 |
| 2-3 | 0.88 | 0.37 | | 2.08 |  |
| 3-4 | 0.90 | 0.39 | | 2.12 |  |
| >4 | 1.09 | 0.42 | | 2.87 |  |
| PQ treatment | 1.16 | 0.73 | | 1.83 | 0.526 |
| Mixed Pf/Pv | 0.32 | 0.17 | | 0.58 | <0.001 |
| First Pf infection | 0.45 | 0.25 | | 0.81 | 0.007 |
| Days post treatment (ref: 0-60) | | |  | |  |
| 61-120 | 1.33 | 0.63 | | 2.81 | 0.299 |
| 121-180 | 1.40 | 0.67 | | 2.94 |  |
| >180 | 0.79 | 0.35 | | 1.78 |  |
| Constant | 0.65 | 0.23 | | 1.83 | 0.417 |

**Table S1.5 Category counts of the GEE Models for *P. falciparum* gametocyte positivity with categorical *Pf* blood-stage densities (genomic 18S rRNA copies/µl). NOTE:** The increase of *Pf* gametocyte positivity with increasing *Pf* asexual density is not very linear because of the initial decrease from the reference category to categories 1-2, 3-4 and >4.

| **Category counts** | *Pf* gametocyte positivity | |  |
| --- | --- | --- | --- |
| *Pf* density category  log_10_(copyno) | 0 | 1 | Total |
| <1 | 40 | 17 | 57 |
| 1-2 | 114 | 32 | 146 |
| 2-3 | 106 | 55 | 161 |
| 3-4 | 69 | 51 | 120 |
| >4 | 47 | 24 | 71 |
| Total | 376 | 179 | 555 |

**Table S1.6 GEE Models for *P. falciparum* gametocyte density (*pfs25* transcripts/µl) with categorical (red) *Pf* blood-stage densities (genomic 18S rRNA copies/µl). NOTE:** Association of categorical *Pf* densities with *Pf* gametocyte densities is not very linear, but *Pf* density is anyways not at all significant neither as linear nor categorical. For simplicity, linear was chosen.

| ***Pf* gametocyte density – categorical *Pf density*** | OR | 95% CI |  | p-value |
| --- | --- | --- | --- | --- |
| Pf density (ref: log10 density <1) | | |  |  |
| 1-2 | 0.77 | 0.48 | 1.24 | 0.145 |
| 2-3 | 0.65 | 0.41 | 1.04 |  |
| 3-4 | 0.88 | 0.56 | 1.39 |  |
| >4 | 0.58 | 0.34 | 1.01 |  |
| PQ treatment | 0.84 | 0.66 | 1.08 | 0.176 |
| First Pf infection | 0.96 | 0.68 | 1.36 | 0.825 |
| Mixed Pf/Pv | 0.76 | 0.53 | 1.08 | 0.13 |
| Fever | 0.76 | 0.49 | 1.17 | 0.211 |
| Days post treatment (ref: 0-60) | | |  |  |
| 61-120 | 0.78 | 0.48 | 1.28 | 0.597 |
| 121-180 | 0.74 | 0.44 | 1.24 |  |
| >180 | 0.74 | 0.47 | 1.16 |  |
| Constant | 4.13 | 2.60 | 6.58 | <0.001 |
