# Supplementary material for: Effects of liver-stage clearance by Primaquine on gametocyte carriage of Plasmodium vivax and P. falciparum
Source: PLoS Negl Trop Dis. 2017 Jul 21;11(7):e0005753. doi: 10.1371/journal.pntd.0005753 (PMC5540608; doi:10.1371/journal.pntd.0005753)
Supplement: S1 Fig — A. and C. Pv and Pf gametocyte densities were expressed as log10 of pvs25 and pfs25 transcripts/μl. B. and D. Pv and Pf parasite densities were expressed as log10 of pv18S rRNA and pf18S rRNA gene copies/μl. C. and E. Pv and Pf normalized gametocyte densities. Densities were normalized by division of pvs25 and pfs25 transcripts/μl by pv18S rRNA or Pf18S rRNA genomic copies/μl, respectively. (DOCX) [file pntd.0005753.s002.docx]

# Effects of liver-stage clearance by Primaquine on gametocyte carriage of *Plasmodium vivax* and *P. falciparum*

***Wampfler et al. 2017***

**S1 FIGURE**

 **S1 Figure.**Gametocyte and overall parasite density in submicroscopic and LM-positive *Pv* (top) and *Pf* (bottom) infections. A. and C. *Pv*  and *Pf* gametocyte densities were expressed as log10 of *pvs25* and *pfs25* transcripts/µl. B. and D. *Pv* and *Pf* parasite densities were expressed as log10 of *pv18S rRNA* and *pf18S rRNA* gene copies/µl . C. and E. *Pv* and *Pf* normalized gametocyte densities. Densities were normalized by division of *pvs25* and *pfs25* transcripts/µl by *pv18S rRNA* or *Pf18S rRNA* genomic copies/µl, respectively.
