# Supplementary material for: Effects of liver-stage clearance by Primaquine on gametocyte carriage of Plasmodium vivax and P. falciparum
Source: PLoS Negl Trop Dis. 2017 Jul 21;11(7):e0005753. doi: 10.1371/journal.pntd.0005753 (PMC5540608; doi:10.1371/journal.pntd.0005753)
Supplement: S3 Fig — A. and C. Proportion of P. vivax and P. falciparum gametocyte carriers among subsequent infections by treatment arm. Figures within the bars indicate absolute numbers of gametocyte-positive subsequent infections following treatment. Error bars indicate 95% confidence intervals by X2 distribution. B. and E. Normalized P. vivax and P. falciparum gametocyte densities in subsequent infections by treatment arm. Normalization was done by dividing pvs25 or pfs25 transcript numbers/μl by Pv- or Pf-18S rRNA copy numbers/μl. C. and F. Absolute P. vivax and P. falciparum gametocyte densities in subsequent infections by treatment arm. Densities are expressed as log10 of pvs25 and pfs25 transcripts/μl. (DOCX) [file pntd.0005753.s004.docx]

# Effects of liver-stage clearance by Primaquine on gametocyte carriage of *Plasmodium vivax* and *P. falciparum*

***Wampfler et al. 2017***

**S3 FIGURE**

**S3 Figure**. Gametocyte positivity and density in subsequent (i.e. not first) *P. vivax* (top) and *P. falciparum* (bottom) infections after treatment with blood-stage antimalarials alone (Placebo) or blood-stage antimalarials plus Primaquine (PQ). A. and C. Proportion of *P. vivax* and *P. falciparum* gametocyte carriers among subsequent infections by treatment arm. Figures within the bars indicate absolute numbers of gametocyte-positive subsequent infections following treatment. Error bars indicate 95% confidence intervals by X^2^ distribution. B. and E. Normalized *P. vivax* and *P. falciparum* gametocyte densities in subsequent infections by treatment arm. Normalization was done by dividing *pvs25* or *pfs25* transcript numbers/µl by *Pv-* or *Pf-18S rRNA* copy numbers/µl. C. and F. Absolute *P. vivax* and *P. falciparum* gametocyte densities in subsequent infections by treatment arm. Densities are expressed as log_10_ of *pvs25* and *pfs25* transcripts/µl.
