# Supplementary material for: Effects of liver-stage clearance by Primaquine on gametocyte carriage of Plasmodium vivax and P. falciparum
Source: PLoS Negl Trop Dis. 2017 Jul 21;11(7):e0005753. doi: 10.1371/journal.pntd.0005753 (PMC5540608; doi:10.1371/journal.pntd.0005753)
Supplement: S1 Table — (DOCX) [file pntd.0005753.s005.docx]

# Effects of liver-stage clearance by Primaquine on gametocyte carriage of *Plasmodium vivax* and *P. falciparum*

***Wampfler et al. 2017***

**S1 TABLE**

**S1 Table. Multivariate risk factors of *P. vivax* and *P. falciparum* asexual parasite positivity during follow-up. Positivity was assessed by *Pv-* or *Pf-18S rRNA* qPCR.**

|  | *P. vivax* parasite positive | | | | *P. falciparum* parasite positive | | | | | | | |
| --- | --- | --- | --- | --- | --- | --- | --- | --- | --- | --- | --- | --- |
|  | OR | 95% CI | | p-value | OR | 95% CI | | | | p-value | |  |
| PQ treatment | 0.14 | 0.09 | 0.22 | <0.001 | 0.90 | 0.61 | | 1.32 | | 0.591 | |  |
| Mixed P.f./P.v. (by qPCR) | 1.82 | 1.33 | 2.49 | <0.001 | 1.83 | 1.38 | | 2.42 | | <0.001 | |  |
| Fever | 0.69 | 0.51 | 0.93 | 0.016 | 3.69 | 2.63 | | 5.17 | | <0.001 | |  |
| Infection at enrolment (same species, qPCR) | 1.38 | 1.05 | 1.81 | 0.023 | 1.93 | 1.25 | | 3.00 | | 0.003 | |  |
| Village |  |  |  |  |  |  | |  | |  | |  |
| Albinama | 1 |  |  | <0.001 | 1 |  | |  | | <0.001 | |  |
| Amahup | 0.29 | 0.18 | 0.47 |  | 0.65 | 0.31 | | 1.34 | |  |  |  |
| Balanga | 1.43 | 0.91 | 2.24 |  | 2.05 | 1.03 | | 4.05 | |  |  |  |
| Balif | 0.72 | 0.47 | 1.11 |  | 0.89 | 0.46 | | 1.73 | |  |  |  |
| Bolumita | 2.30 | 1.53 | 3.48 |  | 9.40 | 4.97 | | 17.80 | |  |  |  |
| Numangu | 0.56 | 0.30 | 1.04 |  | 3.44 | 1.81 | | 6.55 | |  |  |  |
| Age |  |  |  |  |  |  | |  | |  | |  |
| <6y | 1 |  |  | 0.246 | 1 |  | |  | | 0.018 | |  |
| 6-7.5y | 1.07 | 0.72 | 1.58 |  | 1.46 | 0.85 | | 2.51 | |  |  |  |
| 7.6-9y | 0.91 | 0.60 | 1.38 |  | 1.97 | 1.15 | | 3.40 | |  |  |  |
| >9y | 0.73 | 0.47 | 1.14 |  | 2.18 | 1.28 | | 3.72 | |  |  |  |
| Days after DOT |  |  |  |  |  |  | |  | |  | |  |
| 0-60 | 1 |  |  | <0.001 | 1 |  | |  | | <0.001 | |  |
| 61-120 | 1.89 | 1.46 | 2.44 |  | 1.91 | 1.37 | | 2.67 | |  |  |  |
| 121-180 | 1.48 | 1.09 | 2.01 |  | 2.39 | 1.66 | | 3.44 | |  |  |  |
| >180 | 1.20 | 0.89 | 1.62 |  | 2.43 | 1.70 | | 3.48 | |  |  |  |
| Interaction Days after DOT x PQ treatment | | | |  |  | |  | |  | |  | |
| 0-60 x PQ | 1 |  |  |  |  | |  | |  | |  | |
| 61-120 x PQ | 1.47 | 0.84 | 2.55 | 0.175 | not significant | | | | | | | |
| 121-180 x PQ | 2.61 | 1.44 | 4.75 | 0.002 |  |  |  |  |  |  |  |  |
| >180 x PQ | 2.04 | 1.07 | 3.91 | 0.031 |  |  |  |  |  |  |  |  |

OR, odds ratio, DOT, directly observed treatment. ORs were obtained using binomial generalized estimating equations with logit-link allowing for repeated visits by backselection from the full model. The full model included fever, infection status at enrolment by qPCR (*P.f.* or *P.v.* positive), LLIN use (less than 100%), sex, village of residence, hemoglobin at baseline (>9 g/dl). No significant interaction of PQ treatment with days post DOT was detected for *P. falciparum*.
