# Supplementary material for: Effects of liver-stage clearance by Primaquine on gametocyte carriage of Plasmodium vivax and P. falciparum
Source: PLoS Negl Trop Dis. 2017 Jul 21;11(7):e0005753. doi: 10.1371/journal.pntd.0005753 (PMC5540608; doi:10.1371/journal.pntd.0005753)
Supplement: S2 Table — (DOCX) [file pntd.0005753.s006.docx]

# Effects of liver-stage clearance by Primaquine on gametocyte carriage of *Plasmodium vivax* and *P. falciparum*

***Wampfler et al. 2017***

**S2 TABLE**

**Table S2.1 Multivariate risk factors of *P. vivax* gametocyte carriage in subsequent infections during follow-up. Positivity was assessed by *pvs25 rRNA* qPCR.**

| ***Pv* gametocyte positivity** | OR | 95% CI | | p-value |
| --- | --- | --- | --- | --- |
| *Pv* density (10x increase) | 1.88 | 1.32 | 2.68 | <0.001 |
| PQ treatment | 0.94 | 0.56 | 1.56 | 0.797 |
| Mixed Pf/Pv | 0.53 | 0.32 | 0.87 | 0.013 |
| Days post treatment (ref: 0-60) | | |  |  |
| 61-120 | 0.65 | 0.38 | 1.14 | <0.001 |
| 121-180 | 1.51 | 0.82 | 2.80 |  |
| >180 | 2.58 | 1.40 | 4.76 |  |
| Constant | 0.35 | 0.19 | 0.62 | <0.001 |

β, regression coefficient. Coefficients were obtained using Gaussian generalized estimating equations with log-link allowing for repeated visits by back-selection from the full model. The full model included fever, infection status at enrolment by qPCR (*Pf* or *Pv* positive), LLIN use (less than 100%), sex, village of residence, hemoglobin at baseline (>9 g/dl), fever, age. No significant interaction of PQ treatment with days post DOT was detected.

**Table S2.2 Multivariate risk factors of *P. falciparum* gametocyte carriage in subsequent infections during follow-up. Positivity was assessed by *pfs25 rRNA* qPCR.**

| ***Pf* gametocyte positivity** | OR | 95% CI | | p-value |
| --- | --- | --- | --- | --- |
| *Pf* density (10x increase) | 1.40 | 1.07 | 1.84 | 0.016 |
| PQ treatment | 0.90 | 0.49 | 1.63 | 0.721 |
| Mixed Pf/Pv | 0.37 | 0.18 | 0.74 | 0.006 |
| Days post treatment (ref: 0-60) | | |  |  |
| 61-120 | 1.25 | 0.42 | 3.74 | 0.031 |
| 121-180 | 1.06 | 0.37 | 3.05 |  |
| >180 | 0.42 | 0.14 | 1.30 |  |
| Constant | 0.28 | 0.08 | 1.03 | 0.056 |

β, regression coefficient. Coefficients were obtained using Gaussian generalized estimating equations with log-link allowing for repeated visits by back-selection from the full model. The full model included fever, infection status at enrolment by qPCR (*Pf* or *Pv* positive), LLIN use (less than 100%), sex, village of residence, hemoglobin at baseline (>9 g/dl), fever, age. No significant interaction of PQ treatment with days post DOT was detected.
